# The effectiveness of acellular nerve allografts compared to autografts in animal models: A systematic review and meta-analysis

**DOI:** 10.1371/journal.pone.0279324

**Published:** 2024-01-31

**Authors:** Berend O. Broeren, Caroline A. Hundepool, Ali H. Kumas, Liron S. Duraku, Erik T. Walbeehm, Carlijn R. Hooijmans, Dominic M. Power, J. Michiel Zuidam, Tim De Jong

**Affiliations:** 1 Department of Plastic & Reconstructive Surgery, Radboud University Medical Centre, Nijmegen, The Netherlands; 2 Department of Plastic & Reconstructive Surgery, Erasmus MC, Rotterdam, The Netherlands; 3 Department of Plastic, Reconstructive & Hand Surgery, Amsterdam UMC, Amsterdam, The Netherlands; 4 Department of Plastic, Reconstructive & Hand Surgery, Haga Hospital and Xpert Clinic, Den Haag, The Netherlands; 5 Department for Health Evidence Unit SYRCLE, Radboud University Medical Centre, Nijmegen, The Netherlands; 6 Department of Anesthesiology, Pain and Palliative Care (Meta Research Team), Radboud University Medical Centre, Nijmegen, The Netherlands; 7 Department of Hand & Peripheral Nerve Surgery, Queen Elizabeth Hospital, Birmingham, United Kingdom; University College London Institute of Child Health, UNITED KINGDOM

## Abstract

**Background:**

Treatment of nerve injuries proves to be a worldwide clinical challenge. Acellular nerve allografts are suggested to be a promising alternative for bridging a nerve gap to the current gold standard, an autologous nerve graft.

**Objective:**

To systematically review the efficacy of the acellular nerve allograft, its difference from the gold standard (the nerve autograft) and to discuss its possible indications.

**Material and methods:**

PubMed, Embase and Web of Science were systematically searched until the 4th of January 2022. Original peer reviewed paper that presented 1) distinctive data; 2) a clear comparison between not immunologically processed acellular allografts and autologous nerve transfers; 3) was performed in laboratory animals of all species and sex. Meta analyses and subgroup analyses (for graft length and species) were conducted for muscle weight, sciatic function index, ankle angle, nerve conduction velocity, axon count diameter, tetanic contraction and amplitude using a Random effects model. Subgroup analyses were conducted on graft length and species.

**Results:**

Fifty articles were included in this review and all were included in the meta-analyses. An acellular allograft resulted in a significantly lower muscle weight, sciatic function index, ankle angle, nerve conduction velocity, axon count and smaller diameter, tetanic contraction compared to an autologous nerve graft. No difference was found in amplitude between acellular allografts and autologous nerve transfers. Post hoc subgroup analyses of graft length showed a significant reduced muscle weight in long grafts versus small and medium length grafts. All included studies showed a large variance in methodological design.

**Conclusion:**

Our review shows that the included studies, investigating the use of acellular allografts, showed a large variance in methodological design and are as a consequence difficult to compare. Nevertheless, our results indicate that treating a nerve gap with an allograft results in an inferior nerve recovery compared to an autograft in seven out of eight outcomes assessed in experimental animals. In addition, based on our preliminary post hoc subgroup analyses we suggest that when an allograft is being used an allograft in short and medium (0-1cm, > 1-2cm) nerve gaps is preferred over an allograft in long (> 2cm) nerve gaps.

## Introduction

Peripheral nerve injuries affect 2,8% of all trauma cases, and despite surgical repair, they often result in deterioration of quality of life for these patients [1–3]. In some injuries, a segmental loss of a peripheral nerve occurs after trauma or tumor excision for example [4, 5].

The current gold standard for the surgical repair of a peripheral nerve injury that cannot be directly coaptated is a nerve autograft. The sural nerve is the most commonly used because it supplies a consistent source of graft material and is anatomically accessible [6]. However, this procedure has several limitations. The length of donor nerve available and its limited diameter are often insufficient to achieve a complete reconstruction of multiple or significant segmental defects. Besides, the procedure may cause considerable donor site morbidity, such as pain and loss of sensation [7–9]. A specific form of autograft, a vascularized nerve graft, resulted in a superior nerve recovery. However, it could result in an even more considerable donor site morbidity [10].

Several techniques have been investigated to replace the nerve autograft, including allografts, biological conduits and synthetic conduits [11–13]. All with their benefits and drawbacks. Of these options, the acellular allograft seems the most promising [14]. These grafts provide the needed internal structural and molecular composition of the extracellular matrix in a cell-free scaffold, which supports nerve regeneration while retaining a nonimmunogenic nature [15]. This procedure however, has some drawbacks as well, including uncertain histocompatibility and ethical and legal concerns.

A variety of methods have been studied to prepare an acellular allograft such as cold preservation, freeze-thaw cycling, chemical detergent and enzymatic preparations, lyophilization and irradiation [16–18]. There is only one such method that is FDA approved available to surgeons produced by AxoGen, Inc., Alachua, Florida. AxoGen develops its human allografts by combining proprietary detergent processing and gamma irradiation which removes cellular remnants while minimizing the microstructural damage. Next to that, chondroitin sulfate proteoglycan is enzymatic removed from the endoneural tube system to advance axon regeneration [19]. Several additions and alterations to this method have been researched. However, at this moment these techniques are not clinically available.

There is little clinical and experimental evidence about the difference in outcomes between an acellular allograft and an autograft. Therefore, A systematic review of experimental studies was conducted to investigate the efficacy of the acellular nerve allograft, its difference from the gold standard (the nerve autograft) and to discuss the possible indications for the use of an allograft.

## Material and methods

### Research protocol

Before starting this systematic review, a protocol was defined in advance and registered in an international database (PROSPERO, registration number CRD42020186451). The PRISMA guidelines for conducting a systematic review were followed.

### Search strategy

PubMed (Medline), Embase (OVID) and Web of Science were systematically searched to identify all original articles. The search contained studies up to the 4th of January 2022. Search terms included ‘nerve reconstruct’, ‘nerve transfer’, ‘nerve graft’, ‘allograft’, ‘allogeneic’, ‘acellular’, ‘decellularize’ and their synonyms in abstract and title fields (see S1 Table for the complete search strategy). To identify all animal studies, the SYRCLE search filters were used [20, 21]. Endnote (Clarivate Analytics, Pennsylvania, USA) was used to remove duplicates. Two authors (BOB and AK) performed the screening process independently using Rayyan web tool [22]. All titles and abstracts were screened to determine their relevance by utilizing the pre-established inclusion and exclusion criteria. Reference lists of the remaining studies were screened manually for potentially relevant new studies. Full text screening of all relevant articles was done by two reviewers for final selection. Divergences were solved by consensus discussion. Any remaining divergences were solved by consulting TDJ as a third reviewer.

### Inclusion and exclusion criteria

We included an original peer reviewed paper 1) that presented distinctive data; 2) made a clear comparison between not immunologically processed acellular allografts and autologous nerve transfers; 3) was performed in laboratory animals of all species and sex; 4) investigated the effect of acellular allografts on motor outcomes: sciatic function index, muscle weight (gram), ankle angle (degrees), electrophysiology (nerve conduction velocity (ms/s), amplitude (mA) or latency (ms)) and sensory outcomes: hot-cold testing, pin-prick testing, Semmes-Weinstein testing and histomorphometry (axon count and diameter). No publication date restriction was applied.

### Critical appraisal

Two authors (BOB and AK) independently assessed the risk of bias using the SYRCLE’s tool for assessing the risk of bias for animal studies. This appraisal was subsequently merged by consensus and disagreements were solved by discussion [23]. A “yes” indicating a low risk of bias, a “no” indicating a high risk of bias or a “?” indicating an unknown risk of bias was scored for all criteria. We determined selective outcome reporting by establishing if all outcome measures stated in the material and methods section were also reported in the results. Baseline characteristics were: species, age and weight. We included two items to overcome the problem of judging to many items as “unclear risk of bias: reporting on any measure of randomization and reporting on any measure of blinding. For these two questions a “yes” indicates reported and a “no” indicates not reported.

### Data extraction

From the included studies, both reviewers (BOB and AK) extracted the data in duplicate. The descriptive data included: first author’s name, the year of publication, studied species, sex, total number of animals, number of grafts, studied nerve, studied muscle, graft size and time points. The mean, standard deviation (SD) and total number of subjects (n) were recorded for all outcomes. In case multiple locations per nerve were reported, we used the most distal segment of the graft. When the SEM was reported, it was recalculated to SD (SD = SEM x √n). If data were only presented graphically, Universal Desktop Ruler software (https://avpsoft.com/products/udruler/) was used by two reviewers independently to measure a fair estimation of the presented data, after that the mean of these two independent measurements was used. We attempted to contact the authors for additional information in case relevant data were missing.

### Statistical analysis

Comprehensive Meta-Analysis (CMA version 3.3) was used to analyze all data. The standardized mean difference (SMD) and 95% confidence interval (95% CL) for all outcome measurements comparing acellular allografts and conventional autografts were calculated with Hedges’ g correction. A random effects model was applied, which takes the accuracy of independent studies and the variation among studies into account and weighs all studies accordingly. I^2^ was used to asses heterogeneity. In case a study reported results at different time points using the same experimental group, these results were pooled to obtain an overall SMD with Hedges’g correction using a random-effects model and variance. Subgroup analyses were conducted post hoc for species (rat, rabbit, monkey and dog) and graft lengths (0–1 cm, > 1–2 cm and > 2 cm). We only interpreted the results of subgroup analysis when groups consisted of 5 or more individual studies.

To detect publication bias funnel plots were created and evaluated on symmetry using Egger’s regression and Trim and Fill analysis, if there were at least 15 or more independent studies per outcome. We plotted the SMD against a sample size-based precision estimate(1/√(n)), because SMDs may cause funnel plot distortion.

A sensitivity analysis was performed to assess the robustness of our findings. The impact of excluding studies published before 2008 and studies that used animals as their own control was evaluated.

## Results

### Study selection process

**Fig 1:**
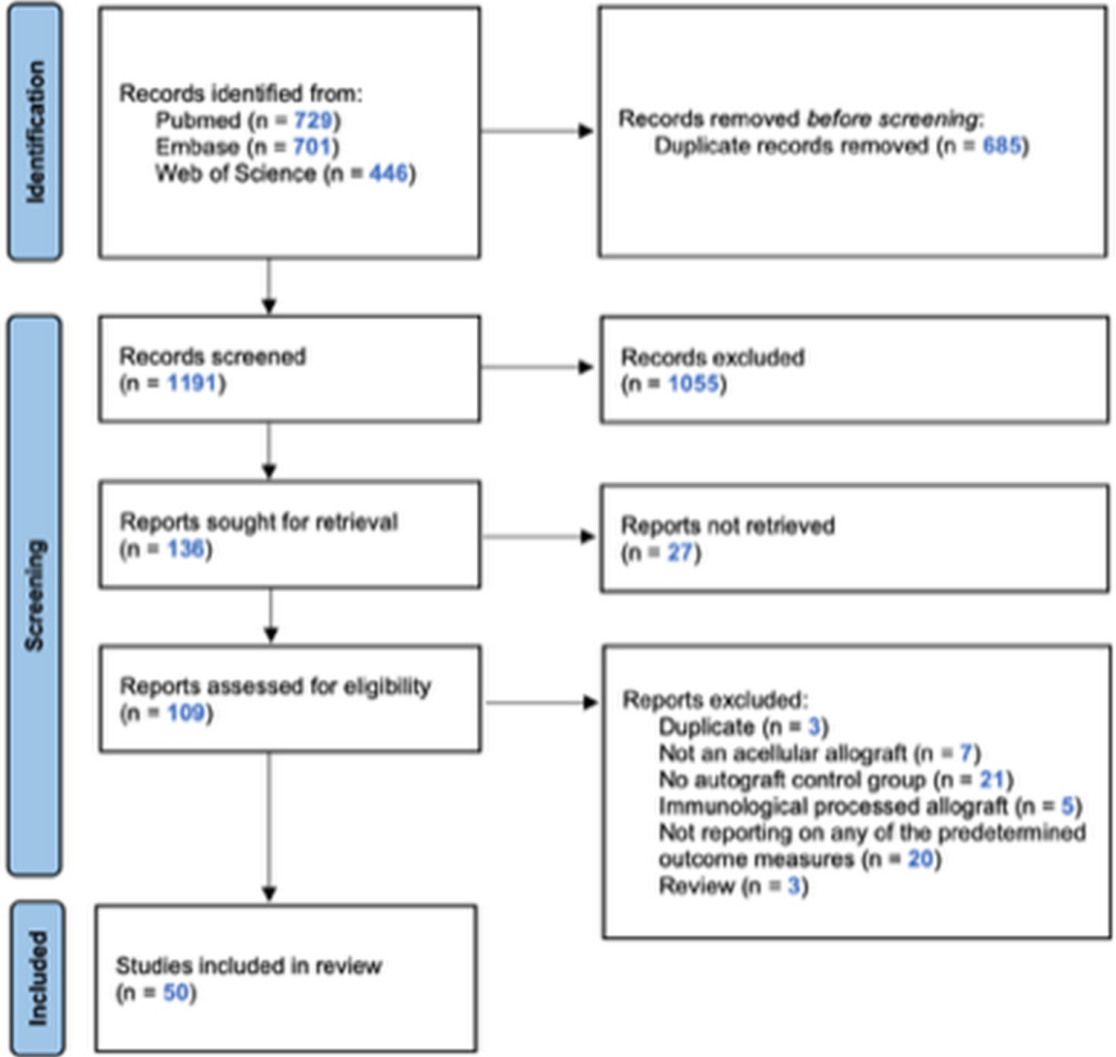
Flow chart of the study selection.

The systematic literature search presented in S1 Table yielded 1191 unique references (Fig 1 shows a consort flow chart). After title abstract screening, 136 studies met the selection criteria. Finally, after studying the full-text articles, 50 studies were included in the review and meta-analyses.

### Study quality and risk of bias

**Fig 2:**
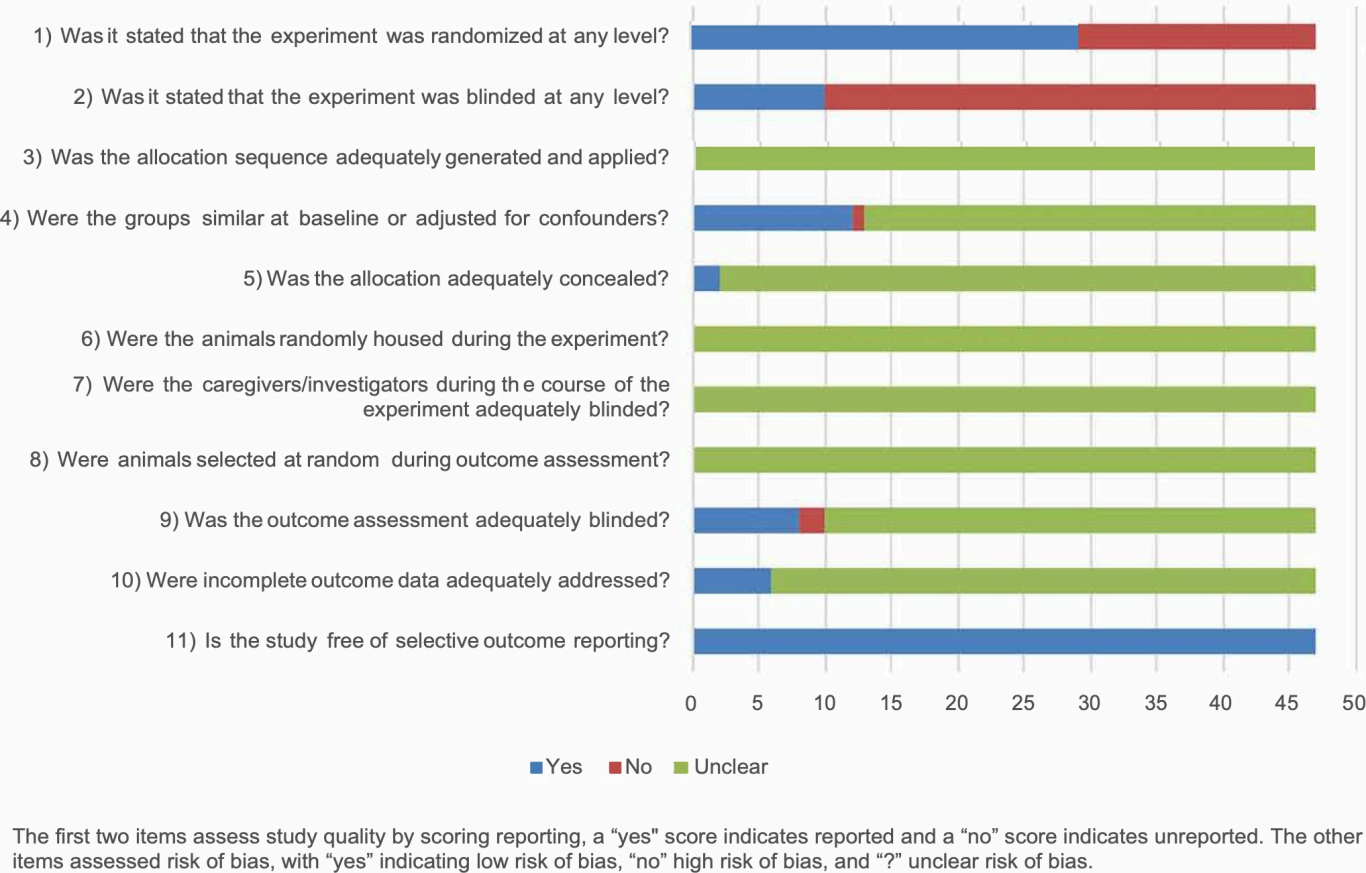
Results of the risk of bias assessment of 47 included studies in this systematic review. The first two items assess study quality by scoring reporting, a “yes” score indicates reported and a “no” score indicates unreported. The other items assessed risk of bias, with “yes” indicating low risk of bias, “no” high risk of bias, and “?” unclear risk of bias.

**Fig 3:**
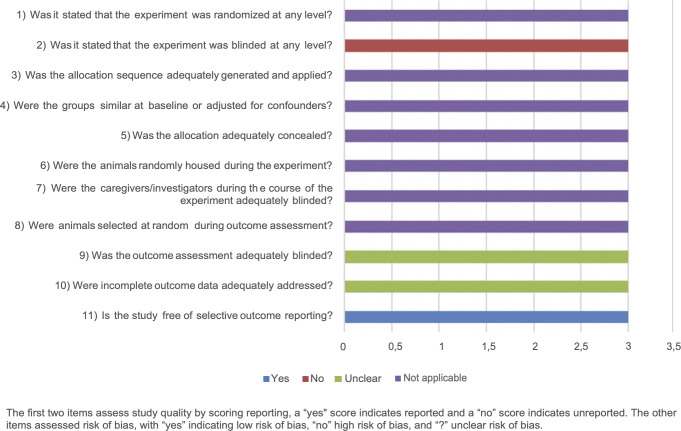
Results of the risk of bias assessment of the 3 included studies in this systematic review where animals were their own control group. The first two items assess study quality by scoring reporting, a “yes” score indicates reported and a “no” score indicates unreported. The other items assessed risk of bias, with “yes” indicating low risk of bias, “no” high risk of bias, and “?” unclear risk of bias.

The general results of our risk of bias assessment of the included references are shown in Fig 2. Poor reporting of essential methodological details in most animal experiments resulted in an unclear risk of bias in most studies. In particular reporting about any randomization and blinding measures taken at any level was 64% (32 out of 50 publications). Assessment of the risk of bias was done separately for the 3 studies that used animals as their own control because some aspects were not applicable (Fig 3).

### Study characteristics

A summary of the characteristics of the 50 included publications is shown in Table 1 [24–73]. The characteristics per outcome measurement are depicted in the Appendix. The most commonly used specie was rat (80%), followed by rabbit (6%), monkey (6%), dog (6%) and mice (2%). Gender was not reported in 20% (10 of 50) of the publications. Out of the remaining studies 33 used males, 2 used females and in 5 both sexes were used. Different nerves were used with the sciatic nerve being the most common (80%), followed by the peroneal nerve (6%), facial nerve (4%), radial nerve (4%), ulnar nerve (2%), tibial nerve (2%) and femoral nerve (2%).

**Table 1:** The characteristics of all 50 included references.

*Reference*	*Species*	*Animals (grafts)*	*Nerve*	*Graft size (mm)*	*Time points (weeks)*	*Outcome measurements*	*Muscle*
Hadlock et al., 2001	Rat	35	Sciatic	7	6, 10.5	SFI	
Boriani et al., 2019	Rabbit	15	Tibial	20	12	NCV Amplitude	
Cai et al., 2017	Rat	18	Sciatic	15	8	Muscle weight Diameter Axonal count	Gastrocnemicus
Chato-Astrain et al., 2020	Rat	24	Sciatic	10	12	SFI Muscle weight	Gastrocnemicus
Dai et al., 2014	Rat	50	Sciatic	10	4, 8, 12	SFI Diameter Axonal count	
Frerichs et al., 2002	Rat	40	Sciatic	20	6	Axonal count	
Gao et al., 2014	Rat	45	Sciatic	10	6, 12	SFI Muscle weight NCV	Gastrocnemicus
Giusti, Lee et al., 2016	Rat	88	Sciatic	10	16	Muscle weight Ankle angle Axon count Tetanic contraction	Tibialis anterior
Giusti, Willems et al., 2012	Rat	65	Sciatic	10	12, 16	Muscle weight Ankle angle Tetanic contraction	Tibialis anterior
Gulati et al., 1990	Rat	24 (48)	Sciatic	20	12	Muscle weight	Extensor digitorum longus
Haase et al., 2003	Rat	19	Peroneal	20, 40	3, 15	Muscle weight Tetanic contraction	Extensor digitorum longus
Huang et al., 2015	Rat	18	Facial	10	12	NCV Diameter Axon count	
Hu, Zhang et al., 2010	Rabbit	72	Facial Peroneal	50	4, 12, 24	Axonal count	
Hu, Zhu et al., 2007	Monkey	12	Ulnar	40	24	NCV	
Hundepool et al., 2018	Rat	66	Sciatic	10	12, 16	Muscle weight Ankle angle Axon count Tetanic contraction	Tibial
Ide et al., 1998	Dog	16	Sciatic	50	12	Axon count	
Jiang et al., 2016	Rat	40	Sciatic	15	12	Muscle weight NCV	Triceps surae
Li, Zhao et al., 2013 [41]	Rabbit	51	Sciatic	30	24	NCV Amplitude	
Li, Peng et al., 2008	Rat	30	Sciatic	10	4, 8, 12, 16	SFI Muscle weight Diameter Axon count	Gastrocnemicus Soleus
Moore et al., 2011	Rat	60	Sciatic	14	6, 16	Muscle weight Tetanic contraction	Extensor digitorum longus
Muheremu et al., 2016	Rat	45	Femoral	5	12	Muscle weight Amplitude Diameter	Quadriceps femoris
Nakamoto et al., 2021	Rat	40	Sciatic	5	3, 6, 12, 14	SFI Diameter Axon count	
Piao et al., 2020	Rat	51	Sciatic	20	24	NCV Amplitude	
Qiu et al., 2020	Dog	15	Sciatic	50	24	NCV Diameter Axon count	
Rovak et al., 2004	Rat	16	Peroneal	20	15	Muscle weight Axon count	Extensor digitorum longus
Saheb et al., 2013	Rat	30	Sciatic	20, 40, 60	10, 20	Muscle weight	Extensor digitorum longus
Shin et al., 2019	Rat	20	Sciatic	10	4, 8, 12, 16	Muscle weight Ankle angle Axon count Tetanic contraction	Tibialis anterior
Sun et al., 2009	Rat	24	Sciatic	10	12	NCV Amplitude	
Tang, Kilic et al., 2013	Rat	54	Sciatic	10	6, 12	Muscle weight Tetanic contraction	Tibialis anterior
Tang, Whiteman et al., 2019	Rat	81	Sciatic	10	12, 16, 20	Muscle weight Diameter Axon count Tetanic contraction	Tibilais anterior
Vasudevan et al., 2014	Rat	24	Sciatic	35	12	Muscle weight Axon count	Gastrocnemicus
Wakimura et al., 2015	Rat	14 (22)	Sciatic	15	24	Amplitude	
Wang, Huang et al., 2014 [56]	Monkey	20	Radial	25	20	NCV	
Wang, Itoh et al., 2016	Rat	15	Sciatic	15	24	Amplitude Diameter	
Wang Liu et al., 2010	Monkey	12	Radial	25	20	Muscle weight NCV	Extensor digitorum longus
Wang, Wu et al., 2016	Rat	20 (40)	Sciatic	20	4, 8, 12	SFI Muscle weight Diameter	Gastrocnemicus Triceps surae
Wang, Zhao et al., 2012	Rat	65	Sciatic	15	12	Muscle weight	Triceps surae
Whitlock et al., 2009	Rat	102	Sciatic	14, 28	6, 12, 22	SFI Muscle weight	Gastrocnemicus
Xiang et al., 2017	Rat	55	Sciatic	15	12	SFI Muscle weight NCV	Gastrocnemicus
Yan et al., 2016	Rat	32	Sciatic	20	8	Muscle weight Axon count Tetanic contraction	Extensor digitorum longus
Yu, Peng et al., 2009	Rat	52	Sciatic	10	16	Muscle weight	Triceps surae
Yu, Wen et al., 2020	Rat	48	Sciatic	10	6, 12	SFI NCV Axon count	
Zhang, Tong et al., 2008	Rat	16	Sciatic	10	12	Muscle weight NCV Amplitude	Tibialis anterior
Zhang, Zhang et al., 2014	Rat	30	Sciatic	10	2, 4, 6, 8	SFI Muscle weight NCV Amplitude	Triceps surae
Zhao1 et al., 2014	Rat	52	Sciatic	15	12	Muscle weight	Triceps surae
Zhao2 et al., 2011	Mice	18	Sciatic	10	2, 4, 6, 8	SFI Muscle weight	Triceps surae
Zhong et al., 2007	Dog	15	Sciatic	50	24	Ankle angle NCV	
Zhou, Zhang et al., 2015	Rat	75	Sciatic	10	4, 16	SFI Muscle weight NCV	Gastrocnemicus
Zhou, He et al., 2014	Rat	72	Sciatic	18	2, 4, 6, 8, 10, 12	SFI NCV Diameter Axon count	
Zhu et al., 2015	Rat	72	Sciatic	18	4	Muscle weight Diameter Axon count	Gastrocnemicus

### Overall analysis

Overall analysis showed a significant lower muscle weight, sciatic function index, tetanic contraction, nerve conduction velocity and smaller ankle angle, axon count diameter after treatment with an acellular allograft compared to an autograft (Shown in Table 2). No significant difference in amplitude was found.

**Table 2:** Summary of the overall analyses.

Outcome measurement	SMD (Hedges g)	95% convidence interval	*I* ^2^	No. of comparisions	No. of studies
Muscle weight	-2.39	-1.85 to -2.93	86%	45	31
Sciatic function index	-1.59	-0.44 to -2.73	93%	15	14
Tetanic contraction	-0.54	-0.13 to -0.95	62%	14	9
Ankle angle	-0.98	-0.28 to -1.69	74%	7	5
Nerve conduction velocity	-2.20	-1.66 to -2.75	73%	19	18
Amplitude	-0.79	0.11 to -1.69	83%	9	9
Axon count	-1.40	-0.78 to -2.01	86%	27	18
Diameter	-1.10	-0.32 to -1.89	82%	13	11

### Subgroup analyses

Subgroup analysis of all subgroups containing a minimum of 5 comparisons revealed a significant difference in muscle weight when comparing graft length between acellular allografts and conventional nerve autografts. Autografts showed a more favorable result in long grafts (> 2 cm) than in medium and short grafts (0–1 cm, > 1–2 cm) compared to acellular allografts (see Table 3).

**Table 3:** Subgroup analysis for graft length.

Outcome measurement	SMD (Hedges g)	95% convidence interval	P-value	No. of comparisions
Muscle weight				
• Short vs. long	-2.13 vs. -4.21	-1.33 to -2.94 vs. -2.87 to -5.55	0.045	18 vs. 9
• Medium vs. long	-1.92 vs. -4.21	-1.09 to -2.75 vs. -2.87 to -5.55	0.026	18 vs. 9
Nerve conduction velocity				
• Short vs. long	-2.36 vs. -1.92	-1.49 to -3.23 vs. -0.88 to -2.95	1	8 vs. 6
• Medium vs. long	-2.33 vs. -1.92	-1.23 to -3.43 vs. -0.88 to -2.95	1	5 vs. 6
Axon count				
Short vs. long	-1.46 vs. -1.21	-0.54 to -2.38 vs. -0.08 to -2.33	1	13 vs. 9
Medium vs. long	-1.62 vs. -1.21	-0.14 to -3.11 vs. -0.08 to -2.33	1	5 vs. 9

However, for nerve conduction velocity and axon count no significant difference was found comparing graft length between acellular allografts and conventional nerve autografts.

All other subgroup analyses on graft length could not be interpreted because groups consisted of fewer than 5 studies. The same goes for all subgroup analyses for species.

### Sensitivity analysis

Exclusion of the studies published before 2008 did not alter our results significantly (see S2 Table). Also, when the studies were excluded in which animals were their own control no significant changes were found, only the amplitude SMD improved significantly (0.70 to 1.00), in favor of autografts (S3 Table).

Conclusions of all subgroup analyses appeared to be robust.

### Publication bias analysis

**Fig 4:**
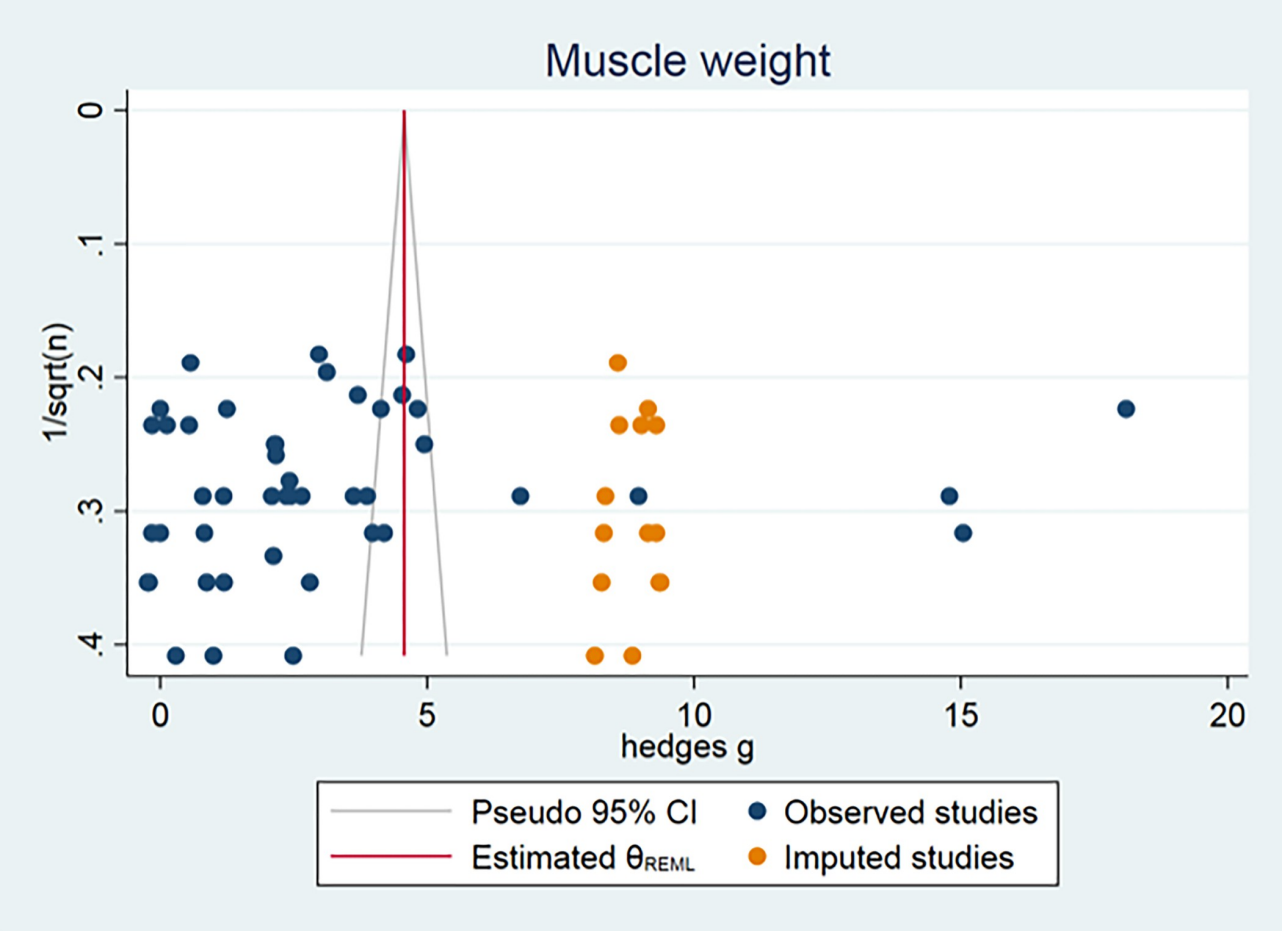
Muscle weight publication bias.

**Fig 5:**
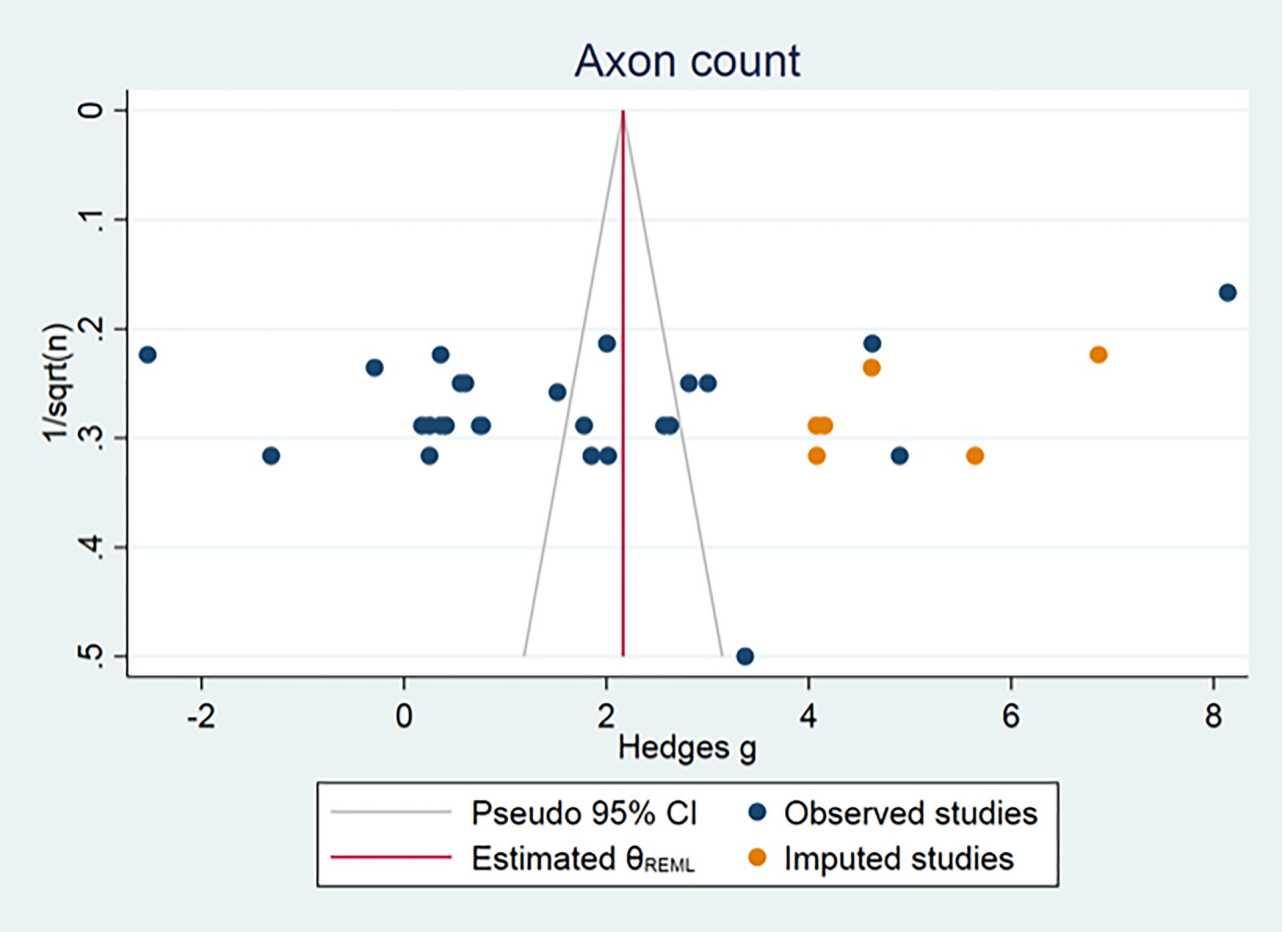
Axon count publication bias.

**Fig 6:**
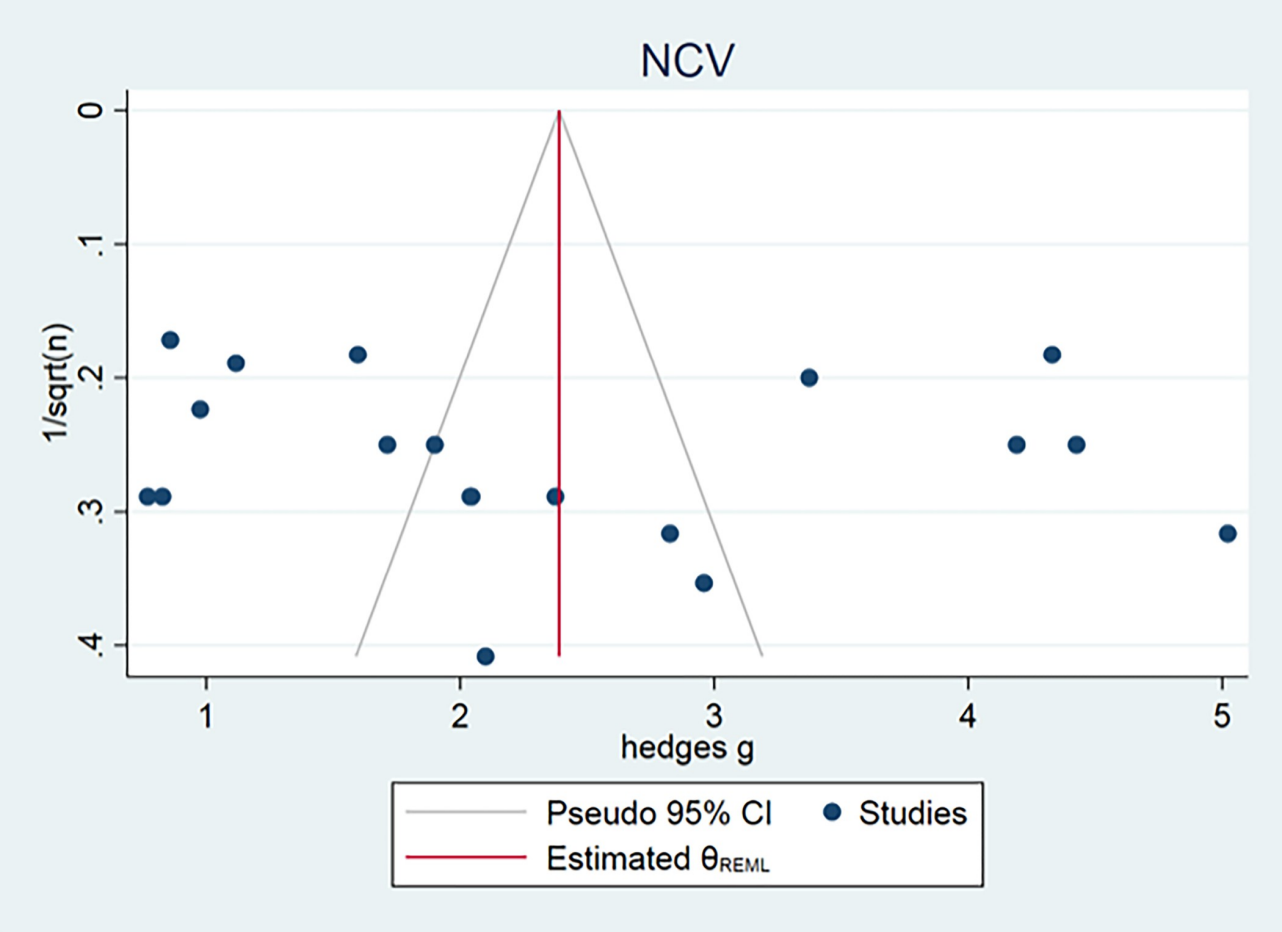
Nerve conduction velocity publication bias.

Publication bias could only be assessed for axonal count, muscle weight and nerve conduction velocity, because all other outcome measurements consisted of fewer than 15 independent studies. The funnel plot for muscle weight and axon count suggested some asymmetry. Duval and Tweedie’s Trim and Fill analysis resulted in 14 and 6 extra data points (Figs 4 and 5), indicating the presence of publication bias and some overestimation of the identified summary effect size. No publication bias for nerve conduction velocity was found (Fig 6).

## Discussion

Our results indicate that treating a nerve gap with an allograft results in an inferior nerve recovery compared to an autograft in seven out of eight outcome measurements assessed in animal models. Our subgroup analysis suggests that when an allograft is being used, an allograft in short and medium (0-1cm, > 1-2cm) nerve gaps performs better than an allograft in long (> 2cm) nerve gaps.

**Fig 7:**
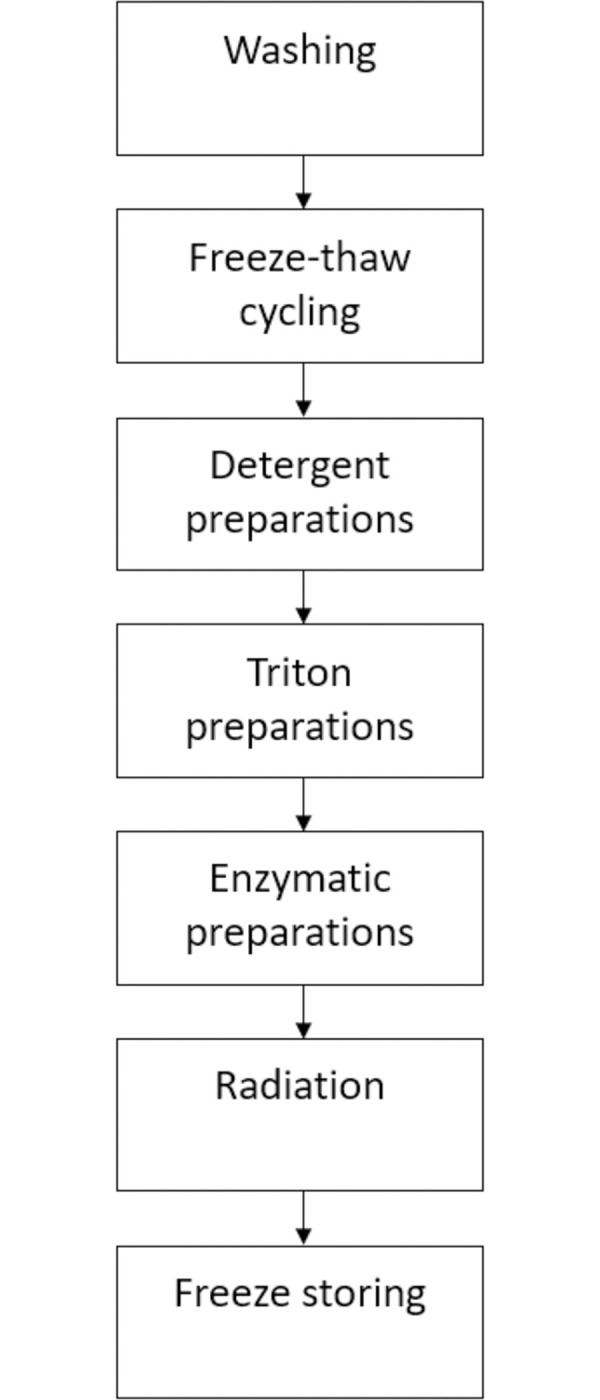
Global overview decellularization methods.

Comparing the available literature regarding the use of acellular allografts was challenging because a large variation in decellularization techniques were used. A variety of methods have been studied to prepare an acellular allograft by these labs such as cold preservation, freeze-thaw cycling, chemical detergent and enzymatic preparations, lyophilization and irradiation [16–18]. There is little to no evidence for what combination of these decellularization methods, give the best nerve regeneration. Fig 7 shows an approximate overview of the different aspects of these methods. We tried to investigate which method led to the best nerve recovery by using the data available in the current literature. Due to the great variation in methods used, groups became too small to perform statistical analysis.

At the moment, there is only one such method that is FDA approved and available to surgeons. It is produced by AxoGen, Inc., Alachua, Florida. AxoGen develops its human allografts by combining proprietary detergent processing and gamma irradiation which removes cellular remnants while minimizing the microstructural damage. Next to that, chondroitin sulfate proteoglycan is enzymatic removed from the endoneural tube system to advance axon regeneration [19]. We noticed that the studies we included, published before 2008, used a minimal decellularization method like mere freeze-thaw cycling. In conclusion, based on the available data and analysis we did, no clear statement could be made as to which decellularization method is superior.

A few human studies show a resemblance in effect between the acellular allograft and the autograft. This would be a significant development because the allograft has a couple of fundamental advantages as opposed to the autograft. It has an unlimited supply that offers an excellent solution, e.g., plexus surgery after a major trauma. In such trauma, there can be insufficient autograft material to repair the nerve deficits. Therefore, in these cases the allograft offers a solution to restore the damages done. It also avoids potential donor site morbidity such as pain and loss of sensation. Next to that, there is the benefit of a shorter operation time. And finally, the off-the shelf availability of the allograft.

The clinical use of commercially available human acellular nerve allografts (AxoGen) for nerve reconstruction has been reported in several case reports and in a more sizeable multicenter study (RANGER) [14, 74–78]. Unfortunately, the multicenter study lacks the opportunity to say anything about the effectiveness of the allograft, because it did not compare it to an autograft.

Safa et al. [77] and Leckenby et al. [78] both reported data from the RANGER study. Safa et al. conducted an analysis of 365 patients with 624 nerve repairs (AxoGen). They found a meaningful sensible and motor function recovery in 82% of cases. Finally, the authors stated that nerve defects up to 7 cm could achieve a useful recovery after treatment. Leckenby et al. analyzed 171 nerve repairs (AxoGen) in 129 subjects exploring a meaningful sensory and motor function recovery as well. In 73.7% of their cases a meaningful sensory recovery (^3^S3) was achieved. The percentage of meaningful motor recovery (^3^M3) was lower at 40,1%, respectively.

Neubauer et al. [19] observed another interesting aspect. They investigated which type of acellular nerve, i.e. motor, sensory or mixed type, is best used for repairing particular types of nerve gaps. No significant differences were found when comparing these three decellularized nerve types as a dominant grafting with regard to axon count and myelinated axons. Most repaired nerves happened to be a sensory dominant one in this study. We noticed that in the animal experiment the opposite is used. The use of motor types was rather common.

Until this day there is no proper “gold standard” to test nerve recovery, although the ultimate goal of nerve recovery is to maximize sensation and motion. The most commonly used outcome measurement for sensation is the von Frey test [79]. For motion, walking track analysis was believed to be the best overall assessment [80–82]. It is rarely used and some would say it is even obsolete. Additionally, walking track analysis does not reflect maximum muscle force capacity. Others say the most precise measurement is the isometric response of muscle to tetanic contraction [83]. The authors are aware that histomorphometry, electrophysiology and axonal count in particular may have a limited correlation to the real functional recovery of sensation and strength [84]. Next to that, histomorphometry is difficult to compare between different laboratories, because other methods to measure the outcome were used. We used a standardized mean difference for our meta-analysis to compensate for these differences. Over the years methods have evolved from manually calculating axonal count from a light microscopic photograph to a computer calculated estimate. The methods used by the studies in this review vary as well. Searching the publication databases, we found little evidence on which one is the best or on a clear sensitivity or specificity for these methods. However, Kim et al. [85] concluded that the semi-automated method for counting axons in transmission electron microscopic images was strongly correlated with conventional counting methods and showed excellent reproducibility. Nevertheless, the techniques for histomorphometry will always be an estimation and therefore prone to bias.

### Limitations of this review

Firstly, the risk of bias analysis revealed that many essential methodological details were poorly reported in the majority of included studies, which is why most risk of bias items assessed in this analysis were scored as ‘unclear risk of bias’. Drawing reliable conclusions from the included animal studies may have hampered the reliability of the analyses presented in this review.

Secondly, for some outcome measurements the number of included studies in this meta-analysis is relatively low, as a consequence the results of these small meta analyses may be imprecise. Next to that, the heterogeneity between the studies was moderate to high. We used a random effects model, subgroup analyses and conducted two different sensitivity analyses to account for this anticipated heterogeneity.

## Conclusion

This review demonstrates that an acellular nerve allograft results in a significantly inferior nerve recovery compared to an autograft in animal models. In addition, when an allograft is being used an allograft in short and medium (0-1cm, > 1-2cm) nerve gaps performs better than an allograft in long (> 2cm) nerve gaps. However, the several different animal experiments, investigating the use of acellular allografts, are difficult to compare due to the wide variety of study designs used and the generally poor reporting of essential methodological details. Large population studies or comparative clinical trials with large study populations to allow for participant and injury heterogeneity will be needed to prove and improve the success of the acellular allograft. We strongly advise future animal studies to be designed and reported according to the ARRIVE guidelines [86, 87].

## Supporting information

S1 Checklist PRISMA 2020 checklist. (PDF)

S1 Table Search strategy. (DOCX)

S2 Table Sensitivity analyses for exclusion of the studies published before 2008. (DOCX)

S3 Table Sensitivity analysis for exclusion of studies in which animals were their own control. (DOCX)

S1 File Raw data. (XLSX)
