# Supplementary material for: The effectiveness of acellular nerve allografts compared to autografts in animal models: A systematic review and meta-analysis
Source: PLoS One. 2024 Jan 31;19(1):e0279324. doi: 10.1371/journal.pone.0279324 (PMC10829984; doi:10.1371/journal.pone.0279324)
Supplement: S1 Table — (DOCX) [file pone.0279324.s002.docx]

**S1 Table. Search strategy.**

| **PubMed**  ("Nerve Transfer"[Mesh] OR "Peripheral Nerves/transplantation"[Mesh] OR Nerve graft*[tiab] OR Nerve transplant*[tiab] OR nerve transfer*[tiab] OR Nerve reconstruct*[tiab] OR Neural transplant*[tiab] OR Neural transfer*[tiab] OR Neural reconstruct*[tiab]) AND (Allograft [Mesh] OR Allograft [tiab] OR Allografts [tiab] OR Allogeneic graft* [tiab] OR Allogenic graft* [tiab] OR Allogeneic [tiab] OR Allogenic [tiab] OR Acellular* [tiab] OR Decellularize* [tiab] OR decellularize* decellular* or allogeneic transplant*, OR homograft* OR homologous transplant*) AND (Experimental animal filter (1)) |
| --- |
| **Embase**  ((nerve reconstruction/ or exp nerve transplantation/ OR exp peripheral nervous system/su) OR ((Nerve graft* OR Nerve transplant* OR nerve transfer* OR Nerve reconstruct* OR Transfer nerve* OR Neural graft* OR Neural transplant* OR Neural transfer* OR Neural reconstruct*).ti,ab,kw.)) AND ((Allograft OR Allogeneic OR Allogenic OR Acellular OR Decellularize* OR decellularize* decellular* or allogeneic transplant* OR homograft* OR homologous transplant*).ti,ab,kw.) AND (Experimental animal filter (2)) |
| **Web of Sience**  (“Nerve graft*” OR “Nerve transplant*” OR “nerve transfer*” OR “Nerve reconstruct*” OR “Transfer nerve*” OR “Neural graft*” OR “Neural transplant*” OR “Neural transfer*” OR “Neural reconstruct*”) AND (Allograft* OR Allogeneic* OR Allogenic* OR Acellular* OR decellularize* OR decellular* or “allogeneic transplant*” OR homograft* OR homologous transplant*) AND (Experimental animal filter)) |

1. Hooijmans CR, Tillema A, Leenaars M, Ritskes-Hoitinga M. Enhancing search efficiency by means of a search filter for finding all studies on animal experimentation in PubMed. Lab Anim. 2010;44(3):170-5.

2. de Vries RB, Hooijmans CR, Tillema A, Leenaars M, Ritskes-Hoitinga M. A search filter for increasing the retrieval of animal studies in Embase. Lab Anim. 2011;45(4):268-70.
