# Supplementary material for: The effectiveness of acellular nerve allografts compared to autografts in animal models: A systematic review and meta-analysis
Source: PLoS One. 2024 Jan 31;19(1):e0279324. doi: 10.1371/journal.pone.0279324 (PMC10829984; doi:10.1371/journal.pone.0279324)
Supplement: S2 Table — (DOCX) [file pone.0279324.s003.docx]

**S2 Table. Sensitivity analyses for exclusion of the studies published before 2008.**

| **Outcome measurement** | **SMD (Hedges g)** | **95% convidence interval** | **I ^2^** | **No. of comparisions** | **No. of studies** |
| --- | --- | --- | --- | --- | --- |
| Muscle weight | -2.32 | -1.76 to -2.88 | 86% | 41 | 27 |
| Sciatic function index | -1.58 | -0.36 to -2.8 | 94% | 14 | 13 |
| Tetanic contraction | -0.34 | -0.01 to -0.67 | 40% | 12 | 8 |
| Ankle angle | -1.09 | -0.32 to -1.87 | 77% | 6 | 4 |
| Nerve conduction velocity | -2.29 | -1.70 to -2.88 | 75% | 17 | 16 |
| Axon count | -1.36 | -0.69 to -2.04 | 87% | 24 | 15 |
