# Supplementary material for: The effectiveness of acellular nerve allografts compared to autografts in animal models: A systematic review and meta-analysis
Source: PLoS One. 2024 Jan 31;19(1):e0279324. doi: 10.1371/journal.pone.0279324 (PMC10829984; doi:10.1371/journal.pone.0279324)
Supplement: S3 Table — (DOCX) [file pone.0279324.s004.docx]

**S3 Table. Sensitivity analysis for exclusion of studies in which animals were their own control.**

| **Outcome measurement** | **SMD (Hedges g)** | **95% convidence interval** | **I ^2^** | **No. of comparisions** | **No. of studies** |
| --- | --- | --- | --- | --- | --- |
| Muscle weight | -2.58 | -2.01 to -3.15 | 86% | 42 | 29 |
| Sciatic function index | -1.67 | -0.43 to -2.91 | 94% | 14 | 13 |
| Amplitude | -1.00 | -0.07 to -1.94 | 82% | 8 | 8 |
| Diameter | -1.28 | -0.46 to -2.11 | 81% | 12 | 10 |
