# Supplementary material for: The effectiveness of acellular nerve allografts compared to autografts in animal models: A systematic review and meta-analysis
Source: PLoS One. 2024 Jan 31;19(1):e0279324. doi: 10.1371/journal.pone.0279324 (PMC10829984; doi:10.1371/journal.pone.0279324)
Supplement: Attachment — Submitted filename: Respons to Reviewer.docx [file pone.0279324.s006.docx]

**Rebuttal letter**

PONE-D-22-33060
“The effectiveness of acellular nerve allografts compared to autografts in animal models: a systematic review and meta-analysis”

Berend O. Broeren^1*^, Caroline A. Hundepool^2^, Ali H. Kumas^1^, Liron S. Duraku^3^, Erik T. Walbeehm^4^, Carlijn R. Hooijmans^5,6^, Dominic M. Power^7^, J. Michiel Zuidam^2^, Tim De Jong^1^

PLOS ONE

Panayiotis Maghsoudlou

Academic Editor

PLOS ONE

Dear Mr Maghsoudlou,

We thank you and the reviewer for a careful reading and the constructive comments regarding our manuscript and for the opportunity to revise and resubmit. We have addressed all recommendations and suggestions to further improve the manuscript. On behalf of my co-authors, I thank you for considering this revised manuscript for publication. We appreciate your time and look forward to your response.

Yours sincerely,

Berend Broeren (corresponding author)

[berend.broeren@xs4all.nl](mailto:berend.broeren@xs4all.nl)

Journal Requirements:

When submitting your revision, we need you to address these additional requirements.

1. Please ensure that your manuscript meets PLOS ONE's style requirements, including those for file naming. The PLOS ONE style templates can be found at

<https://journals.plos.org/plosone/s/file?id=wjVg/PLOSOne_formatting_sample_main_body.pdf> and

<https://journals.plos.org/plosone/s/file?id=ba62/PLOSOne_formatting_sample_title_authors_affiliations.pdf>

2. We note that this manuscript is a systematic review or meta-analysis; our author guidelines therefore require that you use PRISMA guidance to help improve reporting quality of this type of study. Please upload copies of the completed PRISMA checklist as Supporting Information with a file name “PRISMA checklist”.

3. Thank you for stating the following in the Competing Interests section:

“Conflict of Interest Liron S. Duraku and Dominic M. Power:

Both authors are on the educational committee of the Global Nerve Foundation which is a non-profit organization of which Axogen is a founding Member. Both authors do not get any financial compensation for their role in the Global Nerve Foundation.”

Please confirm that this does not alter your adherence to all PLOS ONE policies on sharing data and materials, by including the following statement: "This does not alter our adherence to  PLOS ONE policies on sharing data and materials.” (as detailed online in our guide for authors <http://journals.plos.org/plosone/s/competing-interests>).  If there are restrictions on sharing of data and/or materials, please state these. Please note that we cannot proceed with consideration of your article until this information has been declared.

Please include your updated Competing Interests statement in your cover letter; we will change the online submission form on your behalf.

**The cover letter was changed and the conflict of interest section was added and extended.**

4. Please review your reference list to ensure that it is complete and correct. If you have cited papers that have been retracted, please include the rationale for doing so in the manuscript text, or remove these references and replace them with relevant current references. Any changes to the reference list should be mentioned in the rebuttal letter that accompanies your revised manuscript. If you need to cite a retracted article, indicate the article’s retracted status in the References list and also include a citation and full reference for the retraction notice.

**The reference list is complete and correct**

Comments to the Author

1. Is the manuscript technically sound, and do the data support the conclusions?

The manuscript must describe a technically sound piece of scientific research with data that supports the conclusions. Experiments must have been conducted rigorously, with appropriate controls, replication, and sample sizes. The conclusions must be drawn appropriately based on the data presented.

Reviewer #1: Yes

2. Has the statistical analysis been performed appropriately and rigorously?

Reviewer #1: Yes

3. Have the authors made all data underlying the findings in their manuscript fully available?

The [PLOS Data policy](http://www.plosone.org/static/policies.action#sharing) requires authors to make all data underlying the findings described in their manuscript fully available without restriction, with rare exception (please refer to the Data Availability Statement in the manuscript PDF file). The data should be provided as part of the manuscript or its supporting information, or deposited to a public repository. For example, in addition to summary statistics, the data points behind means, medians and variance measures should be available. If there are restrictions on publicly sharing data—e.g. participant privacy or use of data from a third party—those must be specified.

Reviewer #1: Yes

4. Is the manuscript presented in an intelligible fashion and written in standard English?

PLOS ONE does not copyedit accepted manuscripts, so the language in submitted articles must be clear, correct, and unambiguous. Any typographical or grammatical errors should be corrected at revision, so please note any specific errors here.

Reviewer #1: Yes

5. Review Comments to the Author

Please use the space provided to explain your answers to the questions above. You may also include additional comments for the author, including concerns about dual publication, research ethics, or publication ethics. (Please upload your review as an attachment if it exceeds 20,000 characters)

Reviewer #1: The article is a systematic review and meta-analysis of "The efficacy of acellular nerve allografts compared to autografts in animal models".
This is a very interesting topic of study and I believe that the authors have done a great job of searching for information from many papers as demonstrated in the Flow Chart shown in Figure 1 and the literature provided.
I would like to congratulate the researchers for the great work done with this systematic review and meta-analysis as it will serve many other researchers in this field of peripheral nerve regeneration.

**We appreciate the positive comments of the reviewer.**

I have suggestions for minor changes that I would have to make to the researchers:
1. As this is a systematic review and meta-analysis, I understand that the authors have followed the PRIMA guidelines, but they should specify this in the methodology as they make no mention of these guidelines.

**We have added a mention about the fact that the PRISMA guidelines were followed. Page 5 line 108, 109**

2. I believe that the order of figures 1 and 2 is wrong, as the first time the figures are cited, figure 2 appears first (page 4) and not figure 1 (page 8).

**We have changed the order of figures, figure 2 has become figure 7 on page 14**.

3. Have you considered studying in more detail the decellularizations protocols used for this type of treatment? From your point of view, and after the study carried out, what would you consider to be the best decellularization protocol or technique?

**We think this is a fair question and it is one we asked ourselves as well. However, there is little to no evidence for what combination of decellularization methods, give the best nerve regeneration. We tried to investigate which method led to the best nerve recovery by using the data available in the current literature. Due to the great variation in methods used, groups became too small to perform statistical analysis.**

**We added information on how the authors think about the question on page 14, 15 line 264-269**
